# Complete Genome Sequence of the d-Amino Acid Catabolism Bacterium *Phaeobacter* sp. Strain JL2886, Isolated from Deep Seawater of the South China Sea

**DOI:** 10.1128/genomeA.00913-16

**Published:** 2016-09-01

**Authors:** Yingnan Fu, Rui Wang, Zilian Zhang, Nianzhi Jiao

**Affiliations:** State Key Laboratory of Marine Environmental Science, Institute of Marine Microbes and Ecospheres, Xiamen University, Xiamen, People’s Republic of China

## Abstract

*Phaeobacter* sp. strain JL2886, isolated from deep seawater of the South China Sea, can catabolize d-amino acids. Here, we report the complete genome sequence of *Phaeobacter* sp. JL2886. It comprises ~4.06 Mbp, with a G+C content of 61.52%. A total of 3,913 protein-coding genes and 10 genes related to d-amino acid catabolism were obtained.

## GENOME ANNOUNCEMENT

In the marine environment, the d-amino acids synthesized by microbes can be released into the seawater (1). d-Amino acids (d-AAs), as the α-carbon enantiomers of l-amino acids (l-AAs), are commonly known as nonproteinogenic amino acids (2), and there are few reports about marine bacteria utilizing d-amino acids as carbon and nitrogen sources (3). A bacterial strain, JL2886, was isolated from deep seawater at 2,000-m depth of South China Sea collected during a cruise organized by the National Natural Science Foundation of China in August 2012. Phylogenetic analysis based on the 16S rRNA gene sequences revealed that strain JL2886 belongs to the genus *Phaeobacter*, *Roseobacter* clade (4). JL2886 can utilize many d-AAs as a sole source of carbon or nitrogen for growth (our unpublished data).

The complete genome sequencing of strain JL2886 was performed using the PacBio RS platform (Pacific Biosciences). A 10-kb library was sequenced using P4-C2 chemistry on two single-molecule real-time (SMRT) cells. The average read length was 6,734 bp, with a sequencing depth of 289×. The continuous long reads (CLR) were assembled *de novo* using SMRT Analysis version 2.1 and the protocol PacBio Hierarchical Genome Assembly Process (HGAP) (5). The consensus polishing process resulted in a highly accurate self-overlapping contig, as observed using Gepard dotplot (6), with a length of 4,061,725 bp, in addition to five self-overlapping 678,758-bp plasmids, and the overall G+C content of strain JL2886 was 61.52%. DNA methylation was determined using the RS Modification and Motif Analysis protocol within the SMRT Portal version 1.3.3.

The genome was annotated using Prodigal version 2.6 (7), RNAmmer version 1.2 (8), and ARAGORN version 1.2 (9), as implemented in the Prokka automatic annotation tool version 1.11 (10). The main chromosome contained 12 rRNA operons, 58 tRNAs, a predicted 3,913 protein-coding genes, and 744 protein-coding genes in plasmids.

The predicted open reading frames (ORFs) were annotated through comparisons with the NCBI-NR database and KEGG protein database (11). The functional genes were then identified by association with Clusters of Orthologous Groups (COGs) classification (12) and the KEGG pathway collection (13). A total of 3,690 proteins matched to known functions in the genome. There were 2,641 proteins classified to COG categories and 2,120 proteins classified to KEGG orthologs.

The genome sequences from strain JL2886 contained 361 predicted protein-coding sequences (CDSs) related to amino acid transport and metabolism, including five CDSs for putative d-AA transferases, four CDSs for putative d-AA racemase, and one CDS for putative d-AA oxidases. Strain JL2886 has robust d-AA catabolism ability.

### Accession number(s).

The data from this complete genome sequence have been deposited at DDBJ/EMBL/GenBank under accession no. CP015124. The version described in this paper is the first version, CP015124.1.

## References

[1] Kawasaki N, Benner R 2006 Bacterial release of dissolved organic matter during cell growth and decline: molecular origin and composition. Limnol Oceanogr 51:2170–2180. doi:10.4319/lo.2006.51.5.2170.

[2] Radkov AD, Mcneill K, Uda K, Moe LA 2016 d-Amino acid catabolism is common among soil-dwelling bacteria. Microbes Environ 31:165–168 doi:10.1264/jsme2.ME15126. 27169790 PMC4912152

[3] Kubota T, Kobayashi T, Nunoura T, Maruyama F, Deguchi S 2016 Enantioselective utilization of d-amino acids by deep-sea microorganisms. Front Microbiol 7:511. doi:10.3389/fmicb.2016.00511. 27148200 PMC4836201

[4] Thole S, Kalhoefer D, Voget S, Berger M, Engelhardt T, Liesegang H, Wollherr A, Kjelleberg S, Daniel R, Simon M, Thomas T, Brinkhoff T 2012 *Phaeobacter gallaeciensis* genomes from globally opposite locations reveal high similarity of adaptation to surface life. ISME J 6:2229–2244. doi:10.1038/ismej.2012.62. 22717884 PMC3504968

[5] Koren S, Schatz MC, Walenz BP, Martin J, Howard JT, Ganapathy G, Wang Z, Rasko DA, McCombie WR, Jarvis ED, Phillippy AM 2012 Hybrid error correction and *de novo* assembly of single-molecule sequencing reads. Nat Biotechnol 30:693–700. doi:10.1038/nbt.2280. 22750884 PMC3707490

[6] Krumsiek J, Arnold R, Rattei T 2007 Gepard: a rapid and sensitive tool for creating dotplots on genome scale. Bioinformatics 23:1026–1028. doi:10.1093/bioinformatics/btm039. 17309896

[7] Hyatt D, Chen GL, Locascio PF, Land ML, Larimer FW, Hauser LJ 2010 Prodigal: prokaryotic gene recognition and translation initiation site identification. BMC Bioinformatics 11:119. doi:10.1186/1471-2105-11-119. 20211023 PMC2848648

[8] Lagesen K, Hallin P, Rødland EA, Staerfeldt H-H, Rognes T, Ussery DW 2007 RNAmmer: consistent and rapid annotation of ribosomal RNA genes. Nucleic Acids Res 35:3100–3108. doi:10.1093/nar/gkm160. 17452365 PMC1888812

[9] Laslett D, Canback B 2004 ARAGORN, a program to detect tRNA genes and tmRNA genes in nucleotide sequences. Nucleic Acids Res 32:11–16. doi:10.1093/nar/gkh152. 14704338 PMC373265

[10] Seemann T 2014 Prokka: rapid prokaryotic genome annotation. Bioinformatics 30:2068–2069. doi:10.1093/bioinformatics/btu153. 24642063

[11] Kanehisa M, Araki M, Goto S, Hattori M, Hirakawa M, Itoh M, Katayama T, Kawashima S, Okuda S, Tokimatsu T, Yamanishi Y 2008 KEGG for linking genomes to life and the environment. Nucleic Acids Res 36:D480–D484. doi:10.1093/nar/gkm882. 18077471 PMC2238879

[12] Marchler-Bauer A, Zheng C, Chitsaz F, Derbyshire MK, Geer LY, Geer RC, Gonzales NR, Gwadz M, Hurwitz DI, Lanczycki CJ, Lu F, Lu S, Marchler GH, Song JS, Thanki N, Yamashita RA, Zhang D, Bryant SH 2013 CDD: conserved domains and protein three-dimensional structure. Nucleic Acids Res 41:D348–D352. doi:10.1093/nar/gks1243. 23197659 PMC3531192

[13] Moriya Y, Itoh M, Okuda S, Yoshizawa AC, Kanehisa M 2007 KAAS: an automatic genome annotation and pathway reconstruction server. Nucleic Acids Res 35:W182–W185. doi:10.1093/nar/gkm321. 17526522 PMC1933193

